# Discerning Fragmentation Dynamics of Tropical Forest and Wetland during Reforestation, Urban Sprawl, and Policy Shifts

**DOI:** 10.1371/journal.pone.0113140

**Published:** 2014-11-19

**Authors:** Qiong Gao, Mei Yu

**Affiliations:** Department of Environmental Sciences and Institute for Tropical Ecosystem Studies, University of Puerto Rico, Rio Piedras, San Juan, PR, United States of America; DOE Pacific Northwest National Laboratory, United States of America

## Abstract

Despite the overall trend of worldwide deforestation over recent decades, reforestation has also been found and is expected in developing countries undergoing fast urbanization and agriculture abandonment. The consequences of reforestation on landscape patterns are seldom addressed in the literature, despite their importance in evaluating biodiversity and ecosystem functions. By analyzing long-term land cover changes in Puerto Rico, a rapidly reforested (6 to 42% during 1940–2000) and urbanized tropical island, we detected significantly different patterns of fragmentation and underlying mechanisms among forests, urban areas, and wetlands. Forest fragmentation is often associated with deforestation. However, we also found significant fragmentation during reforestation. Urban sprawl and suburb development have a dominant impact on forest fragmentation. Reforestation mostly occurs along forest edges, while significant deforestation occurs in forest interiors. The deforestation process has a much stronger impact on forest fragmentation than the reforestation process due to their different spatial configurations. In contrast, despite the strong interference of coastal urbanization, wetland aggregation has occurred due to the effective implementation of laws/regulations for wetland protection. The peak forest fragmentation shifted toward rural areas, indicating progressively more fragmentation in forest interiors. This shift is synchronous with the accelerated urban sprawl as indicated by the accelerated shift of the peak fragmentation index of urban cover toward rural areas, i.e., 1.37% yr^−1^ in 1977–1991 versus 2.17% yr^−1^ in 1991–2000. Based on the expected global urbanization and the regional forest transition from deforested to reforested, the fragmented forests and aggregated wetlands in this study highlight possible forest fragmentation processes during reforestation in an assessment of biodiversity and functions and suggest effective laws/regulations in land planning to reduce future fragmentation.

## Introduction

Land use and land cover have been changing dramatically across the globe and significantly impact both natural and human systems [1], [2], [3]. Urbanization, deforestation, and reforestation in abandoned agricultural areas are considered major drivers of the spatial distribution and pattern changes of urban areas, forests, and wetlands in recent decades [2], [4], [5], [6].

Although deforestation as a means to meet the food demands of the growing population has been a major topic [7], [8], [9], [10], [11], [12], reforestation has been significant in regions with an economic shift from agriculture to industry or to service during economic globalization, which is discussed in the Forest Transition Theory [2], [5], [13], [14]. Reforestation via secondary succession may greatly alter the ecosystem composition, structure, and function [15] and thus add uncertainties to the large-scale carbon budget and water dynamics in the context of global change.

Urbanization is often coupled with the reforestation during the economic shift. People migrated from rural to urban areas after the abandonment of agriculture [16]. The global urban population comprised 10% of the total population in the 1900s and is predicted to comprise 60% in 2030 [17]. The rapid urbanization may accelerate the process of reforestation in rapidly developing countries, such as China. Meanwhile, urban areas expanded at twice the rate of the population increase due to rapid suburban development [3]. While the global urban population is predicted to reach 5 billion in 2030 from 2.86 billion in 2000, the projected urban area in 2030 is nearly triple that in 2000 [3]. Urban expansion drives environmental changes at local, regional, and global scales and impacts biodiversity, biogeochemical cycles, hydrology, climate, and ecosystem services [3], [17].

Wetlands are vulnerable to land use/cover changes globally [18]. Because 70% of the global population lives within 80 km of coastlines [19], coastal wetlands are disappearing rapidly due to real estate development and cultivation. Wetlands are further affected by the “coastal squeeze” due to sea level rise [20]. The average annual coastal wetland loss in the US increased from 60,000 acres (24,281 ha) during 1998–2004 to 80,000 acres (32,375 ha) during 2004–2009 [21].

The patterns of land cover changes are often characterized by fragmentation [22], [23]. The extension of urban sprawl to distant suburbs and the deforestation processes have been found to cause landscape fragmentation [7], [24]. Landscape fragmentation is considered one of the major threats to biodiversity and a major driver of changes in ecosystem structure, function, and disturbance regimes [25], [26], [27], [28], [29], [30]. Enhanced edge effects due to fragmentation reduce mass, energy, and information flows within a land type, but increase the exchanges among different land types [31], [32]. The biophysical environment and socioeconomic factors are often used to explain fragmentation dynamics, such as the analysis of urban sprawl in Maryland, US [24].

Compared with a large volume of literature on deforestation and landscape fragmentation [26], [33], [34], the role of reforestation in fragmentation has rarely been investigated. Few studies address the composite impacts of urbanization and reforestation on landscape fragmentation dynamics. Although deforestation is still the dominant topic in the studies of land use land cover changes, economic globalization, intensified agriculture, and environmental protection indicate that reforestation, and often associated urbanization, may become widespread in heavily agriculture-dependent regions in the future. The interactions between urbanization and reforestation and their consequences on landscape fragmentation are especially important in tropical regions that contribute significantly to global biodiversity and carbon sequestration [9], [35].

Puerto Rico, an island in the Caribbean, is an ideal place to investigate the coupled impacts of urbanization and reforestation on landscape dynamics in tropical regions. Due to the economic shift from agriculture to industry and services, Puerto Rico has been experiencing significant urbanization, reforestation, and urban sprawl since the 1940s [16]. Forest cover increased from 6% of Puerto Rico in the 1940s to 42% in 2000. Urban areas increased from 1.7% in the 1950s to 11% in 1990 and to 14% in 2000, following the global trend of urbanization [17], [36]. The human population doubled during this period and reached 3.73 million in 2010 (US Census 2010). The population density of 425 people km^−2^ is greater than that in all 50 US states except for New Jersey. A trend of urban-to-suburban migration (urban sprawl) was revealed [13], [37] with the expansion of the low-density residential area from 1991 to 2000, which may fragment natural ecosystems [24]. On the other hand, secondary succession transforms abandoned croplands to pastures, shrublands, and forests [13], [38]. Reforestation may favor the aggregation of forests and thus reduce fragmentation. The composite impacts of the two drivers, viz. reforestation and urban sprawl, determine the changes in the patterns of natural systems, e.g., forests and wetlands. However, the quantitative relationship between changes in landscape fragmentation and the composite drivers is still unknown.

Coastal wetlands are especially vulnerable at this heavily populated area. Many wetlands were historically drained for sugar cane production [39] and were recently filled for coastal development [13], [40]. The distribution of mangroves declined by 45% during the cultivation era, and coastal urbanization in the 1960s induced a further decrease [41]. The trend reversed in the 1970s due to the policy shift for wetland protection [38]. However, the patterns and underlying mechanisms of the wetland dynamics are still unknown.

Our objectives are to investigate the spatiotemporal patterns of the fragmentation of forests, urban areas, and wetlands in Puerto Rico at local and island-wide scales and to provide insights into the relationship between land cover patterns and the drivers of urban sprawl, reforestation, and policy change. We tested the hypotheses that, despite the overall reforestation, forests were still fragmented by the process of urban sprawl; additionally, the coastal wetlands were aggregated in the study period due to policy changes, e.g., the implementation of The RAMSAR Convention on Wetlands and the US Clean Water Act.

## Methods

### Study Area

**Figure 1:**
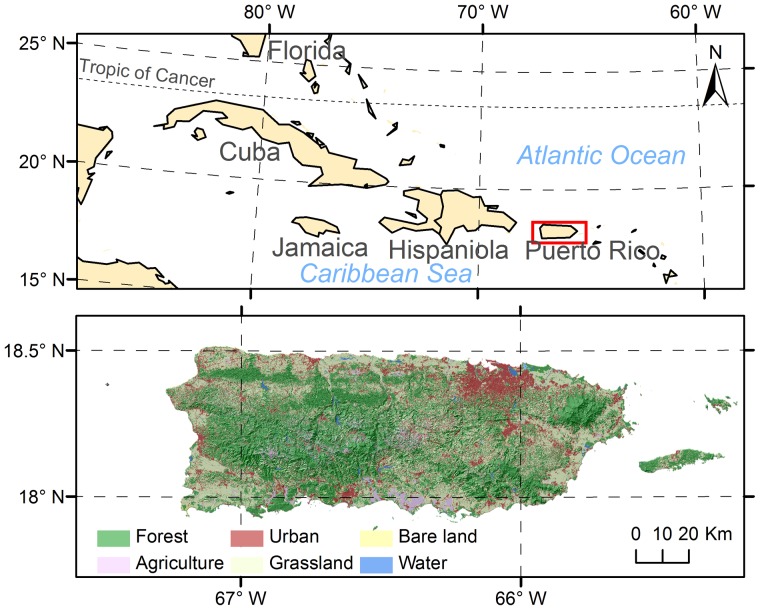
Location, geomorphology, and land cover in 2000 of Puerto Rico (Land cover information from Kennaway and Helmer, 2007).

Our study area is the tropical island of Puerto Rico, which is a US territory that lies at the eastern tip of the Greater Antilles (18°15″N and 66°30″W). Puerto Rico has a total area of 9,104 km^2^ (Fig. 1). The prevailing trade wind comes from the northeast and traverses the central mountain range that extends east-west. The highest peak is 1,338 m. The northern windward side receives much more rainfall than the southern side, resulting in the high spatially-heterogeneous landscapes within the relatively small area. The rainforest at El Yunque Mountain in the northeast receives an annual rainfall of over 4,000 mm, whereas the dry Guanica forest in the south receives less than 800 mm. Approximately 59% of Puerto Rico is subtropical moist forest. Wet forests and rainforests are located in the mountains, and dry forests are mostly located in the southwest.

### Island-wide landscape analysis of urban sprawl, reforestation, and deforestation

To detect the fragmentation dynamics of forests, urban areas, and wetlands, we analyzed the land cover maps for 1977, 1991, and 2000 that were developed by the USDA Forest Service, the Puerto Rico Department of the Natural and Environmental Resources, and the US Geological Survey Caribbean Division [36], [38]. We chose these years due to the map availability, the consistency in the map classification and spatial resolution, and the reflection of urban expansion and forest regrowth [38]. All three maps are at the spatial resolution of 30 m. The maps for 1991 and 2000 were derived from the Landsat TM/ETM images using computerized classification, while the map for 1977 was interpreted visually from the aerial photos [38]. To minimize the effects of different data sources and processing methods, the spatial patterns of fragmentation were compared among all the three years; however, the absolute values of the fragmentation indices were only compared for 1991 and 2000. The original categories of the maps were reclassified into urban, forest, wetland (vegetated), and pasture to singularize the interactions between these categories (File S1). Four landscape indices, the mean patch area (AREA_MN), the edge density (ED), the edge-to-area ratio (EDGE_AREA), and the largest patch index (LPI) were calculated using the FRAGSTAT software [42] for forests, urban areas, and wetlands, respectively (Table 1).

**Table 1:** The fragmentation indices used in the landscape analysis.

Index	Equation	Description
LPI	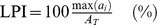	Largest patch index, percentage of the largest patch of the focal type in the landscape, mainly reflecting configuration. *a_i_*, area of the *i^th^* patch (m^2^), *A_T_*, total landscape area (m^2^).
ED	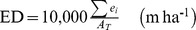	Edge density, edge length of the focal type per hectare of landscape area, reflecting both configuration and composition. *e_i_*, edge of the *i^th^* patch (m).
AREA_MN	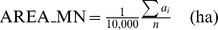	Mean patch area, mainly reflecting configuration. *n*, the number of patches of the focal type.
EDGE_AREA	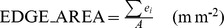	Edge to area ratio, edge length per focal type area, mainly reflecting configuration. *A*, area of the focal type (m^2^).

Most fragmentation indices contain information of both landscape composition and configuration [23], [42]. Generally, ED increases with the number of patches. A larger ED reflects a more fragmented landscape only when the composition of a focal type is fixed. On the other hand, ED becomes a measurement of composition when the patch size and shape are fixed. AREA_MN and EDGE_AREA do not distinguish between a single patch and multiple patches with similar mean sizes and shapes. In fact, AREA_MN only measures the average patch size, and EDGE_AREA measures the average size and shape of the patch(s). These indices mutually compensate each other to measure the fragmentation of landscapes.

To decouple the impacts of urban sprawl on forest fragmentation between 1991 and 2000, the union of the urban areas in both years was used as a mask, and the fragmentation indices of forest were recalculated with the mask for both years. This approach allowed us to assess the forest fragmentation when the effect of urban change was removed. The result was compared to the forest fragmentation with urban changes.

To separate the impacts of reforestation from those of deforestation on forest fragmentation, we first compared the forest distribution of 1991 to that of 2000 to detect the reforestation and deforestation sites. A deforestation-only scenario was then created from the forest distribution of 2000 by eliminating the reforested sites. Similarly, a reforestation-only scenario was formed from the union of the forest distributions in both years (therefore, no deforestation occurred in this scenario). The forest fragmentation indices were calculated for the deforestation-only and the reforestation-only scenarios. The results were compared with those derived from the true forest distributions in 1991 and 2000.

### Spatiotemporal fragmentation analysis at a 3-km scale using spatial error models

To explore the mechanisms of landscape fragmentation dynamics during urban sprawl and reforestation, we aimed to quantify the relationship between the spatiotemporal pattern of fragmentation and biophysical and socioeconomic factors. The biophysical and socioeconomic variables of elevation, slope, distance to urban centers, and population density were considered as the potential causes of fragmentation dynamics. We used a spatial resolution of 30 m for the elevation (www.edc.usgs.gov), the population density (www.census.gov), the slope, and the distance to urban areas in 1977 (created using the Spatial Analyst extension of ArcGIS software, Esri, Redlands, CA).

We divided the main island of Puerto Rico into 1,088 non-overlapping large grid cells of 3 km×3 km such that each large grid cell contained 10,000 pixels of 30 m×30 m. Cells with less than 2,500 land pixels (25% of the whole cell) were discarded, resulting in 1,004 cells in the subsequent analyses. For each large cell, we calculated the above fragmentation indices for urban, forest, and wetland classes for 1977, 1991, and 2000. The averages and standard deviations of the distance to urban centers, elevation, geomorphological slopes, and population density, were also calculated for each large cell. Among these variables, the distance to urban centers and population density were considered the composite variables that reflect socioeconomic status. Slopes, including their heterogeneity, have a strong control on urban development and reforestation.

We used a spatial error model to regress the fragmentation indices of the large cells onto the biophysical and socioeconomic variables. The spatial error model represents a linear relationship between the dependent (*y*) variable and a number of independent variables (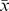),
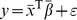
(1)

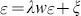
(2)where *y* refers to any of the fragmentation indices, 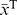 is a transposition of the biophysical and socioeconomic factors, 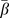 is the vector of coefficients, and *ε* is the error. Unlike an ordinary linear regression model, the spatial error model assumes that the error is spatially correlated, as indicated in Eq. (2), where *λ* is a scalar of the spatial correlation coefficient, *w* is a weight matrix that describes the spatial correlation structure, and *ξ* is the error component without spatial dependence. The *w* matrix is usually derived from the neighborhood, and the eight nearest neighbors are used for this analysis. The spatial regressions were conducted in the R environment (version 3.0.1, package spdep) to estimate 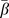, *λ*, and *ξ* [43].

To assess the average changes in the fragmentation over the island, increments of the fragmentation indices from 1991 to 2000 were computed for each large cell, and the spatial error model was applied to fit an intercept-only model to the increments. The results allow us to determine whether there is a significant change in the fragmentation between 1991 and 2000 at the 3-km scale.

We chose to regress EDGE_AREA and ED for urban and ED and AREA_MN for forest classes on the distance-to-urban (*D*), the mean and standard deviation of the slopes (*s* and 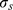), and the mean population density (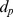). These three indices were chosen because scatterplots of their values versus those of the independent variables showed relatively concentrated patterns. The regressions were intended to derive the spatiotemporal relationships between the landscape fragmentation and the major drivers. Retention of the independent variables in the regression was guided by the minimum Akaike Information Criterion (AIC). Due to the strong topographic restriction on the distribution, we did not attempt to apply such regression to wetlands.

To investigate the interactions of urbanization/urban sprawl and reforestation, we regressed the increments of the three fragmentation indices for urban and forest classes and the EDGE_AREA for the wetland class between 1991 and 2000 onto the changes in the total areas of the different land cover types and the biophysical/socioeconomic variables. The analysis allowed us to assess how the changes in the composition of a particular land cover type affect the fragmentation pattern of various land cover types.

## Results

### Fragmentation analysis at the island-wide scale

Although both forests and wetlands experience total area increase from 1991 to 2000 (both approximately 2%), the island-wide patterns of the two land cover types showed a distinctively different trend: forests are becoming more fragmented, but wetlands are becoming more aggregated (Table 2). The mean forest patch size (AREA_MN) decreased and the forest ED and EDGE_AREA increased, showing increased forest fragmentation. However, the mean wetland patch size more than doubled and the wetland ED and EDGE_AREA decreased, indicating a dramatic reduction in wetland fragmentation.

**Table 2:** Island-wide fragmentation analyses for urban, forest, and wetland areas in 1991 and 2000.

	AREA_MN	LPI	ED	EDGE_AREA
Urban				
1991	1.184	1.39	25.9	415.6
2000	1.182	1.44	28.1	417.7
Wetland				
1991	3.54	0.08	1.76	246.3
2000	7.63	0.09	1.31	185.5
Forest				
1991	8.03	10.3	37	169.2
2000	7.56	14.1	39	174.8
Forest outside urban areas				
1991	7.20	10.4	29	182.6
2000	7.12	14.2	31	181.3
Forest scenarios				
Deforestation only	6.07	7.8	35	196.4
Reforestation only	10.73	19.2	36	136.3

AREA_MN, mean patch area (ha); LPI, largest patch index (%); ED, edge density (m ha^−1^); EDGE_AREA, edge to area ratio (m m^−2^).

The number of urban patches increased during 1991–2000 with an 8% increase in total area. Although the largest urban patch increased, the AREA_MN decreased and the EDGE_AREA and ED increased, which indicated slight urban sprawl (Table 2). The land cover transition showed that as to the new urban/suburban areas in 2000, 55% of them were pastures and 31% were forests in 1991.

The impact of urbanization/urban sprawl on forest fragmentation is quantified by comparing the scenarios of ‘forests’ and ‘forests without urban changes’ (Table 2). The AREA_MN of forests without urban changes slightly decreased from 7.2 to 7.12 ha between 1991 and 2000, which is much smaller than the 6% decrease observed when urban changes were imposed. Although the urban changes dominated the forest fragmentation, a slight trend of forest fragmentation was detected even without urbanization/urban sprawl.

The analysis of forest transitions revealed that approximately 82% of the forest in 1991 remained in 2000 with the other 18% deforested, mostly to pastures and urban areas. Reforestation also occurred, which largely transformed pastures and low-density urban areas into secondary forests. The combined outcome of deforestation (754 km^2^) and reforestation (836 km^2^) resulted in a 2% increase in forests during this period.

**Figure 2:**
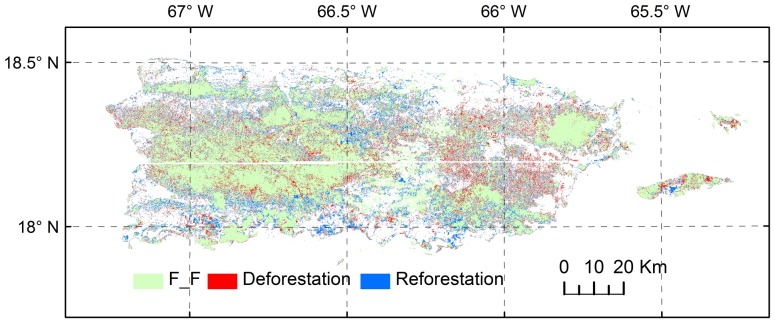
Spatial distributions of reforestation sites, deforestation sites, and unchanged forests (F_F) during the period of 1991–2000. The forest distribution in the deforestation-only scenario includes the F_F only, while that in the reforestation-only scenario includes all the three types. The true distribution in 1991 includes deforestation sites and unchanged forests; the true distribution in 2000 includes unchanged forests and reforestation sites.

The spatial configuration of deforested and reforested sites (Fig. 2) indicated that the deforested sites tend to be located in the forest interiors, while the reforested sites tend to be located along the edges of the forests. The average distance of the reforested sites to the unchanged forests (forests in 1991 remained in 2000) is 66.7 m, while the average distance of the deforested sites to the unchanged forests is 55.8 m (16.3% closer to the interior of the forests compared to the reforested sites).

The fragmentation indices of the deforestation-only and reforestation-only scenarios revealed that the AREA_MN for the deforestation-only scenario is 6.07 ha, which is smaller than that of the true forest scenarios in 1991 and 2000 (i.e., 8.03 and 7.56 ha, respectively). However, the AREA_MN is much higher for the reforestation-only scenario (10.73 ha, Table 2). Deforestation decreased the AREA_MN by 24.4%, but reforestation increased it by 33.6%. The coupled deforestation and reforestation processes (the true forest scenario in 2000) resulted in a decrease of 5.9%, indicating a stronger role of deforestation in the forest fragmentation than that of reforestation due to their different spatial configurations. The same pattern occurred for the index of EDGE_AREA (Table 2), which increased by 16.1% due to deforestation, decreased by 19.4% due to reforestation, but still showed a 3% increase when the two processes were coupled.

### Spatiotemporal pattern of landscape fragmentation at the 3-km scale

**Figure 3:**
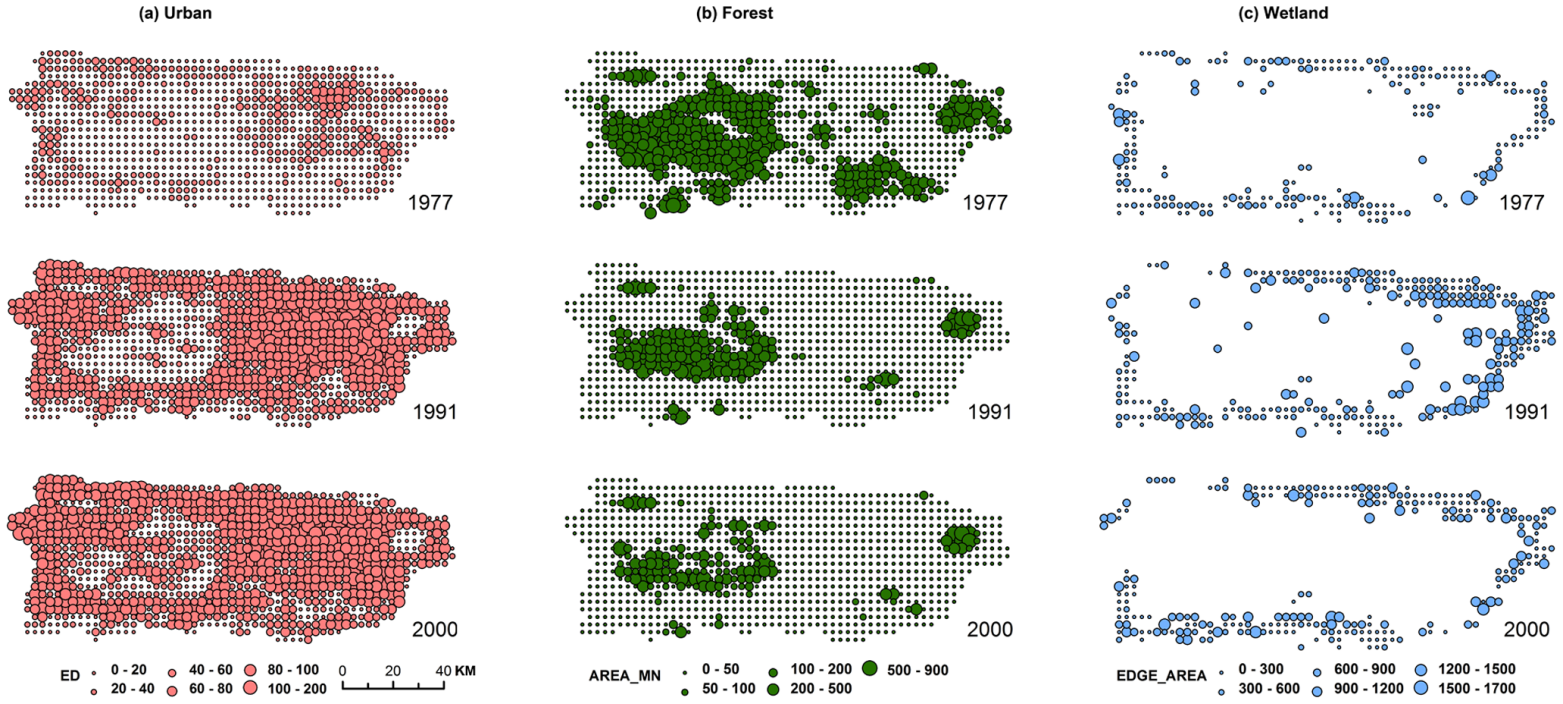
Fragmentation dynamics of (a) urban edge density (ED, m ha^−1^), (b) forest mean patch area (AREA_MN, ha), and (c) wetland edge-to-area ratio (EDGE_AREA, m m^−2^) in 1977, 1991, and 2000.

The fragmentation indices showed different spatial and temporal patterns for urban, forest, and wetland areas (Fig. 3). The fragmentation of urban cover is low within the cities' expansion and tends to be high at the suburban areas, indicating urban sprawl (Fig. 3a). In contrast, forest fragmentation tends to be low in the rural areas, such as the central mountains (Fig. 3b). In general, urban sprawl and forest fragmentation increased from 1991 to 2000, i.e., ED increased and AREA_MN decreased; however, the decreased wetland fragmentation during 1991–2000, especially in the eastern part of the island, indicates wetland aggregation (Fig. 3c) and agrees with the island-wide analysis.

The fitted intercepts of the increments of the fragmentation indices from 1991 to 2000 (Table 3) further confirmed that the enhanced ED (significant for forests and urban areas) and reduced AREA_MN (significant for forests) are strong indicators of increased urban sprawl and forest fragmentation. The results suggest a general trend that a small number of large patches of urban and forest in the large cells may become more aggregated, as reflected in the increased LPI and decreased EDGE_AREA. However, small and moderate patches were more numerous and became more fragmented, causing the reduced AREA_MN and increased ED. The fragmentation of forests is especially prominent, as seen by the 43% decrease in AREA_MN. The significantly reduced ED, increased LPI, and increased AREA_MN (49%) highlight the wetland aggregation.

**Table 3:** Assessment of average increments of the fragmentation indices from 1991 to 2000 by fitting the intercept of the spatial error model.

Fragmentation index		Urban	Forest	Wetland
AREA_MN	Intercept	−0.2	−22.2**	3.4**
	Relative change	−0.08	−0.43	0.49
LPI	Intercept	0.73**	0.57*	0.42*
	Relative change	0.10	0.016	0.10
EDGE_AREA	Intercept	−8.5*	−10.3	−17.0
	Relative change	−0.01	−0.04	−0.03
ED	Intercept	3.8*	3.3*	−6.19**
	Relative change	0.07	0.04	−0.18

‘**’ and ‘*’ indicate the levels of significance with *p* values less than 0.01 and 0.05, respectively. Relative changes are calculated by dividing the intercept by the mean fragmentation indices of 1991 and 2000.

### Relationships between landscape fragmentation and biophysical and socioeconomic drivers

**Figure 4:**
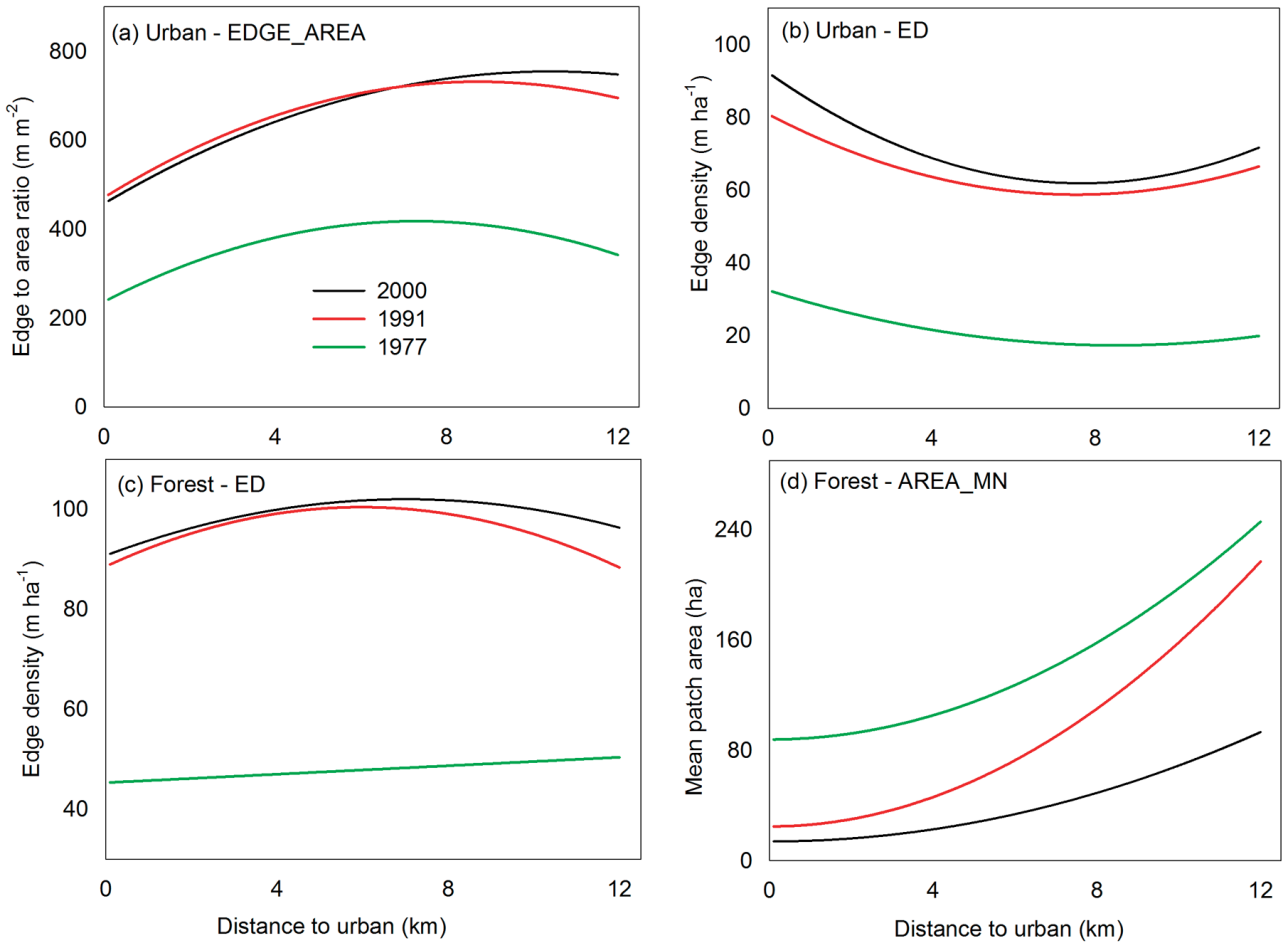
Predicted changes in urban and forest fragmentation index with the distance to urban centers in 1977, 1991, and 2000. (a) the edge-to-area ratio of urban areas; (b) the edge density of urban areas; (c) the edge density of forests; and (d) the mean patch area of forests.

The best-fit spatial error models for the urban EDGE_AREA produced a quadratic function of the distance to urban centers, *D*; the index increased with the slope, *s*, and decreased with the heterogeneity of the slope, 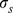, and the population density, *d_p_* (Table 4). The predicted urban EDGE_AREA versus *D* (Fig. 4a) with other variables fixed at their mean values showed that the change from 1977 to 1991 is relatively even across the range of *D*; however, the change from 1991 to 2000 is more complicated. The EDGE_AREA of 2000 is slightly smaller than that of 1991 when *D* is lower than 7.2 km; but greater when *D* is larger. The peaks of the curves shifted from 7.3 in 1977 to 8.7 in 1991 and to 10.4 km in 2000. Because EDGE_AREA is a measure of size and shape, this shift indicates small patches developed progressively further away from the urban centers and implies significant urban sprawl toward suburban and rural areas.

**Table 4:** Spatial error models of land fragmentation indices regressed on biophysical and socioeconomic drivers.

Fragmentation Index	*D*	*D^2^*	*s*	*s^2^*	*σ_s_*	*σ_s_^2^*	*d_p_*	intercept
EDGE_AREA_U,00_	56.9	−2.73	19.98		−38.47		−0.119	455.2
EDGE_AREA_U,91_	59.6	−3.42	20.85		−35.91		−0.121	446.0
EDGE_AREA_U,77_	49.6	−3.41	6.29		−9.31		−0.063	233.5
ED_U,00_	−7.96	0.519	3.58	−0.188	9.18	−1.02	0.00321	54.9
ED_U,91_	−5.87	0.389	3.54	−0.172	5.30	−0.68	0.00463	51.7
ED_U,77_	−3.56	0.21	1.38	−0.066			0.00631	22.8
ED_F,00_	3.20	−0.23	9.34	−0.285	−4.42			40.96
ED_F,91_	3.97	−0.33	10.20	−0.308	−4.71			33.23
ED_F,77_	0.42		4.25	−0.136	−1.87			22.93
AREA_MN_F,00_		0.55	−6.68	0.47	14.45	−1.88		
AREA_MN_F,91_		1.33	−4.41	0.38	12.63	−1.65		
AREA_MN_F,77_		1.10	2.59	0.62	6.93	−2.93		

Values are significant intercepts or coefficients for the corresponding variables. The first subscript of the fragmentation index indicates urban (U) or forest (F), and the second indicates the year of 2000 (00), 1991 (91), or 1977 (77). *D*, distance to urban centers (km); *s*, slope; 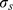, standard deviation of slope; *d_p_*, population density (per km^2^).

The urban edge density decreased with *D*, but increased with slope, slope standard deviation, and population density when these variables are small (Table 4). However, the second order terms of *D*, *s*, and 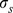, tend to reduce the effects of the first order terms. The maximum ED occurred at *s* of 9.5–10.5° for the three years and at 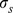 of 3.9–4.5° in 1991 and 2000. The predicted urban ED with *D* (Fig. 4b) indicated that the increase in ED was uniform across the range of *D* from 1977 to 1991. However, ED was more enhanced for smaller *D* than larger *D* from 1991 to 2000. Considering the decreased EDGE_AREA over the small distances, the greater increase in ED means increased large patches in these areas.

Similar to the case of EDGE_AREA for urban areas, the ED of forest increases with the first order of *D* and *s*, but decreases with 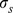 (Table 4). The negative second order term of *D* indicates the existence of a maximum edge density for both 1991 and 2000 (Fig. 4c). When the distance is short, only a few forest patches exist and the ED is low. When the distance is very large, the forest tends to be less fragmented and therefore has fewer edges. These imply a maximum ED in the moderate distances. Indeed, the peak is found at 6.0 and 7.0 km for 1991 and 2000, respectively, singularizing the shift of the maximum forest fragmentation from suburban toward rural areas. Similarly, the negative second-order term of the slope indicates a peak edge density at approximately 15.6–16.5° for the three years.

The effects of these explanatory variables on the forest AREA_MN are opposite those of the ED, except for the lack of the first order term of *D* and an additional term of 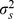 (Table 4). The forest mean patch area increases with 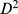, and the curves are all concave upward (Fig. 4d). The AREA_MN consistently decreases from 1977 to 2000 across the range of *D*. However, two different phases are apparent: fragmentation of the forest mostly occurred close to urban areas from 1977 to 1991, as indicated by the great decrease in the AREA_MN for small *D*; further fragmentation from 1991 to 2000 mostly occurred far from urban areas, and the reduction in the AREA_MN from 1991 to 2000 increased substantially with *D*. The AREA_MN also decreases with the first order of *s* for 1991 and 2000 but increases with the first order of 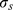 for the three years. The nonlinear effects of both produce a minimum AREA_MN at the slopes of 5.8 in 1991 and 7.1° in 2000 and a maximum AREA_MN at 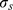 of 3.8° in 2000 and 1991 and of 1.2° in 1977.

### Interactions of the incremental land cover changes and their impacts on the landscape fragmentation

The regression of the changes in the urban EDGE_AREA and ED from 1991 to 2000 yielded the following equations:
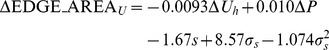
(3)

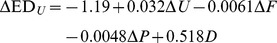
(4)where 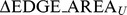 and 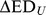 are the incremental changes of urban EDGE_AREA and ED from 1991 to 2000. 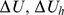, 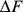, and 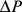 are the corresponding increments of 30 m pixels (proportional to changes in area) for urban, high density urban (class in original land cover maps), forest, and pasture, respectively. Increases in high-density urban and decreases in pasture may both reduce the increment of urban EDGE_AREA. The increase in urban areas may enhance the increment of urban edge density, but increases in forest and pasture tend to reduce the increment of urban edge density.

The increment of the forest edge density (Eq. 5) is enhanced by the increases in urban areas, forests, and pastures, but lowered by the increase in wetlands (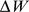, the increase in wetland pixels from 1991 to 2000). On the other hand, the increase in urban area reduces the increment of the forest mean patch area, and the increase in forest pixels tends to enhance the increment (Eq. 6). The incremental EDGE_AREA for wetlands (Eq. 7, 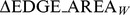) is reduced by the increase in wetlands and the decrease in agricultural land (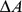).
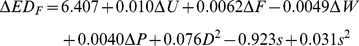
(5)

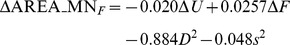
(6)

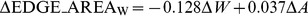
(7)


## Discussion

The deforestation-to-reforestation process of subtropical Puerto Rico is common in many developing regions [44] and supports the Forest Transition Theory [6], [14], [45], [46] with an economic shift from agriculture to industry and service as the main driver. However, our findings indicate that despite the progressively increased forest cover over the course of the reforestation, the forests are becoming more fragmented, primarily due to two drivers: urban sprawl and deforestation in the forest interiors during and after reforestation.

### Accelerated urban sprawl coupled with a forest fragmentation shift to rural areas

The urban sprawl in Puerto Rico is characterized by a shift of the peak edge-to-area ratio from near urban centers to suburban and rural areas and an increased edge density from 1977 to 2000 (Fig. 4, a and b). If we divide the difference between the peaks by the length of the time interval, we find that the average annual peak shift is 0.10 and 0.19 km yr^−1^ for the periods of 1977–1991 and 1991–2000, respectively. The nearly doubled speed of the peak shift in 1991–2000 implies accelerated urban sprawl. It was reported that the peak fragmentation in the state of Maryland, US, shifted from 40 km away from urban centers in 1973 to 55 km away from urban centers in 2000 [24]; the average annual rate was 0.56 km yr^−1^, which is faster than that in Puerto Rico. Considering the difference in the size of land area between Maryland and Puerto Rico, we divided the absolute annual shift by the peak distance of the previous years to compute the relative peak shift rate of 1.39% yr^−1^ for Maryland and 1.37 and 2.17% during 1977–1991 and 1991–2000, respectively, for Puerto Rico. From 1991 to 2000, the peak of the forest edge density shifted from 5.8 to 7.0 km away from urban centers (Fig. 4c); the annual shift was 0.13 km yr^−1^. Hence, the accelerated urban sprawl is coupled and approximately synchronized with the increased forest fragmentation toward rural areas, despite the large increase in forest coverage during 1991–2000. The relationship between urban sprawl and forest fragmentation is also demonstrated in Equations 5 and 6, where increased urban area is shown to cause increased forest edge density and decreased forest mean patch area.

Moreover, the accelerated urban sprawl toward farther distances was also accompanied by urbanization near suburbs. The edge-to-area ratio lacks the ability to discern many small patches from a single small patch; however, the edge density compensates for this. The urban edge density in 2000 is greater than that in 1991 across the entire range of distances to urban centers (Fig. 4b). Hence, this finding is an additional indicator of urban sprawl. However, the increase in the urban edge density is greater at shorter distances than at farther distances due to the increased large urban patches near suburbs, which lowers the edge-to-area ratio and indicates urbanization at shorter distances (Fig. 4a). It is interesting to see that when urbanization (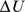) is considered, the increment of the urban edge density from 1991 to 2000 increased with the distance (Eq. 4), as indicated by the positive coefficient in front of *D* in the equation (opposite of the trend displayed in Fig. 4b). In other words, when the change of the urban composition is incorporated into the regression, the changes in the urban edge density mainly reflect changes in the landscape configuration, and the increase in the increment of the edge density with *D* indicates smaller and more irregular urban patches in distant suburbs.

### Forest fragmentation is also caused by deforestation in forest interiors during reforestation

The forest fragmentation is mainly caused by urban sprawl, as indicated by the synchronized shifts of peak fragmentation of both forest and urban cover, and the fact that the increase in the urban area (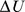) significantly reduced the increment of the forest mean patch area and enhanced the increment of the forest edge density (Eq. 5 and 6). The forest fragmentation analysis with and without urban impacts also supports this finding: a 6% decrease in the forest mean patch area was computed under urban change compared with only a 1% decrease without urban influence. On the other hand, the increase in the forest area (reforestation) enhanced the increments of both the mean patch area and edge density of the forest, implying that reforestation mostly formed large patches (Eq. 5 and 6). Among all the causal factors of the forest edge density increment (Eq. 5), urban development is the strongest, as shown by the greatest coefficient of 0.01 in front of 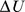, compared with other land type changes.

In addition to urban sprawl, the greater increase in the forest edge density (Fig. 4c) associated with the greater reduction in the mean patch area at farther distances (Fig. 4d) than those at shorter distances to urban areas from 1991 to 2000 consistently signaled forest fragmentation far from urban areas, i.e., at locations closer to the forest interior. For comparison, previous forest fragmentation mostly occurred near urban areas (1977–1991). The distribution of deforestation sites is nearer to the unchanged forests or forest interiors, compared with that of reforestation sites. The island-wide analysis of the deforestation-only and reforestation-only scenarios demonstrates the stronger role of deforestation, compared with reforestation, on forest fragmentation due to its unique spatial configuration.

The deforestation in the forest interior is mostly associated with the development of pastures from 1991 to 2000. About 75% of deforested areas were converted to pastures and 21% were converted to urban areas in 1991–2000. The increase in the forest edge density is positively associated with the increase in the pasture (Eq. 5). Evidence can be easily found in the western part of the central mountains, where forests were converted to many small patches of pasture for various purposes, such as dairy industry development and land for real estate.

### Role of the socioeconomic and biophysical variables in landscape fragmentation

The distance to urban centers, geomorphological slope, and population density played important roles in the urban and forest fragmentation dynamics. The effects of these variables are mostly nonlinear as the second order terms are found in the equations. In a particular year, the fragmentation of forest and urban areas generally increases with the distance to urban centers due to the urban sprawl, reaches a peak, and decreases due to the reduced anthropogenic activities. We found a systematic shift of the peak fragmentation to greater distances, and this trend has been observed in many other places around the world [24].

Slope has been found to be a control variable for both urban and forest areas [24], [47]. Our results showed their fragmentation index increased with gentle to moderate slopes. A steep slope cannot be used to build large urban patches, and the scattered construction further fragments both forests and urban cover. When slopes become too steep, urban development is limited by high costs, low commercial value, and risk of landslides, one of the primary natural hazards in Puerto Rico [48]. Therefore, the forests in steep areas are less likely to be disturbed and form large patches with reduced edge density. The slopes with peaks of urban edge density have been found within the range of 9.5–10.5°. The peak edge density of forests has been found at slopes of 15.6–16.5°, which are higher than those for urban edge density, partly because steeper terrain contains more forests and less urban areas. However, the mean patch area of forests has a minimum at 5.8 and 7.1° for 1991 and 2000, respectively, indicating a slight shift of the fragmentation to steeper terrain; the slopes in these ranges are critically vulnerable to further fragmentation.

The impact of the heterogeneity of the slope, as described in the standard deviation, is seldom discussed in the literature. In this study, when the heterogeneity is small or moderate (within 4°), the increase of the slope standard deviation lowered the edge-to-area ratio but increased the edge density of urban areas. A possible consequence of meeting both conditions is increased large patches of urban areas with slope standard deviations in the range of 0–4°. Within the same range, the forest edge density decreased, but the mean patch area increased. Both findings indicate the decreased forest fragmentation with the heterogeneity of the slope. Beyond this range, the high spatial heterogeneity of the terrain limits urban development and fragments the forests. The shift of the peak mean patch area of forests from 1977 (at 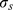 = 1.2) to 1991 and 2000 (at 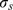 = 3.8) indicates the large forest patches appeared in more heterogeneous areas in 1991 and 2000 than in 1977.

Population density has been shown to be a control variable for urban sprawl, but not for forest fragmentation. The urban edge-to-area ratio decreases with the population density because great population densities are generally associated with large urban patches. However, the urban edge density increases with the population density because large urban patches enhance the edge density in terms of composition. A lack of population density in the equations of the forest fragmentation indices (Table 4) suggests that the role of population density is mostly indirect in forest fragmentation.

### Significant wetland aggregation despite urban sprawl

Unlike the fragmenting dynamics of forests, wetlands are becoming much more aggregated at both island-wide and local scales. However, the spatial variation of wetland fragmentation is independent of changes in either urban areas or forests (Eq. 7) because wetland dynamics are mainly controlled by the implementation of laws and regulations on wetland protection. The international RAMSAR Convention on Wetland, the Clean Water Act by US Congress, and other relevant state regulations have been enforced since the early 1970s [41]. By comparing the distributions of protected areas [49], approximately 44% of wetlands are protected. Specifically, 56% of forested wetlands (e.g., mangroves and *Pterocarpus* forests) and 29% of herbaceous wetlands are protected. The implementation of wetland regulations directly leads to wetland aggregation, as indicated by the greater mean patch area within the reserves (6.8 ha) compared to that outside the reserves (5.1 ha).

A reduction in agriculture led to decreased wetland fragmentation, as indicated in Eq. 7, because the recovery of wetland is mostly from abandoned agriculture, e.g., sugarcane cultivation and other coastal agriculture. As an example, the Caño Tiburones Nature Reserve, which is the largest herbaceous wetland in Puerto Rico, was formed after the pumps used to drain the land for agriculture broke during Hurricane Georges in late September of 1998. The natural recovery of the wetlands was appreciated and a request to establish a nature reserve was approved by law on Oct. 16, 1998. A similar transformation occurred in the largest *Pterocarpus* forest wetland in Puerto Rico.

### Implications of landscape fragmentation during urban sprawl and reforestation

Recent research on global forest cover changes [5] clearly showed that in the period of 2000–2012, deforestation in the forest interiors of Puerto Rico still exists and reforestation continues, especially in the south (map available at http://earthenginepartners.appspot.com/science-2013-global-forest). The global urbanization trend with a faster rate of urban expansion than rate of population increase implies that low-density urban or urban sprawl is continuing in many regions, especially in developing countries, such as China and those in South America [3], [50]. Globalization, intensified agriculture, and economic shifts also caused forest transitions in many areas of the world (i.e., deforestation to reforestation) [6], [51]. Our study indicates that even during the process of reforestation, forests are very likely to become fragmented, primarily due to urban sprawl and deforestation in the forest interiors; the dynamics of forest fragmentation are synchronized with urban sprawl. Because fragmented forests have modified microclimates, disturbance regimes, and biodiversity structures, their functions and services, such as carbon sequestration capabilities, should be evaluated differently from those of continuous forests. Wetlands are becoming aggregated in our study regardless of the strong interference of urbanization along the coast because of the wetland protection regulations. The contrasting trend in fragmentation between forests and wetlands implies that effective regulations and rational land planning shall be implemented for forest protection, especially for the forest interiors.

## Supporting Information

File S1 **Reclassification of the land cover maps for fragmentation analysis.**
(DOCX)
