# Supplementary material for: Discerning Fragmentation Dynamics of Tropical Forest and Wetland during Reforestation, Urban Sprawl, and Policy Shifts
Source: PLoS One. 2014 Nov 19;9(11):e113140. doi: 10.1371/journal.pone.0113140 (PMC4237398; doi:10.1371/journal.pone.0113140)
Supplement: File S1 — Reclassification of the land cover maps for fragmentation analysis. (DOCX) [file pone.0113140.s001.docx]

Reclassification of the land cover maps for fragmentation analysis

For the land cover maps of 1991 and 2000:

Urban – High-Medium Density Urban and Low-Medium Density Urban;

Forest – Evergreen and Seasonal Evergreen Forest on Karst; Evergreen Forest on Serpentine; Elfin, Sierra Palm, Transitional and Tall Cloud Forest; Seasonal Evergreen and Evergreen Forest; Seasonal Evergreen Forest with Coconut Palm; Seasonal Evergreen and Semi-Deciduous Forest on Karst; Drought Deciduous, Semi-deciduous and Seasonal Evergreen Forest on Serpentine; Semi-Deciduous and Drought Deciduous Forest on Karst (includes semi-evergreen forest); Semi-Deciduous and Drought Deciduous Forest on Alluvium and Non-Carbonate Substrates; Deciduous, Evergreen Coastal and Mixed Forest or Shrubland with Succulents; Drought Deciduous Dense Woodland; Drought Deciduous Open Woodland;

Pasture – Pasture, Hay or Inactive Agriculture (e.g. abandoned sugar cane); Pasture, Hay or other Grassy Areas (e.g. soccer fields);

Forested Agriculture – Active Sun Coffee and Mixed Woody Agriculture;

Herbaceous Agriculture – Herbaceous Agriculture - Cultivated Lands;

Forested Wetlands – Mangrove; Seasonally Flooded Savannahs and Woodlands; *Pterocarpus* Swamp;

Herbaceous Wetlands – Emergent Wetlands Including Seasonally Flooded Pasture; Tidally Flooded Evergreen Dwarf-Shrubland and Forb Vegetation;

For the land cover map of 1977:

Urban – Urban and Developed;

Forest – Forest; Woodlands; Shrublands; Vegetation in urban areas;

Pasture – Pasture; Grass;

Forested Agriculture – Coffee; Mixed woody agriculture;

Herbaceous Agriculture – Herbaceous agriculture;

Forested wetlands – Forested wetlands;

Herbaceous wetlands – Non-forested wetland;

For calculating fragmentation index using FRAGSTAT, we used urban and non-urban covers for fragmentation analysis of urban areas, forest and non-forest covers for forest fragmentation analysis, and wetland and non-wetland covers for wetland fragmentation analysis.
